# Клинический случай IgG4-связанного заболевания щитовидной железы у ребенка 6 лет

**DOI:** 10.14341/probl13379

**Published:** 2023-10-18

**Authors:** А. А. Колодкина, Н. А. Зубкова, Л. С. Урусова, С. П. Бондаренко, Д. Н. Бровин, А. В. Аникиев, О. Б. Безлепкина

**Affiliations:** Национальный медицинский исследовательский центр эндокринологии; Национальный медицинский исследовательский центр эндокринологии; Национальный медицинский исследовательский центр эндокринологии; Национальный медицинский исследовательский центр эндокринологии; Национальный медицинский исследовательский центр эндокринологии; Национальный медицинский исследовательский центр эндокринологии; Национальный медицинский исследовательский центр эндокринологии

**Keywords:** тиреоидит Риделя у ребенка, IgG4-связанное заболевание, щитовидная железа

## Abstract

IgG4-связанное заболевание – это редкая хроническая патология, проявляющаяся лимфоплазмоцитарной инфильтрацией одного или нескольких органов, формированием муароподобного фиброза, отеком тканей и повышением IgG4 в крови. Данное заболевание было выделено в самостоятельную нозологическую единицу только в 2001 году. Заболеваемость составляет менее 1 на 100 000 человек в год. При IgG4-связанном заболевании может поражаться практически любой орган. Связь тиреоидита Риделя с IgG4-связанными заболеваниями была установлена в 2010 году. Тиреоидит Риделя является крайне редким воспалительным заболеванием щитовидной железы, особенно у детей. Диагностика осложняется нетипичным течением и отсутствием характерных симптомов. В мире описано менее 300 клинических случаев заболевания, из них всего два у детей. В статье представлен клинический случай тиреоидита Риделя у 6-летнего мальчика.

## Abstract (en)

IgG4-related disease is a rare chronic pathology manifested by lymphoplasmacytic infiltration of one or more organs, the formation of storiform fibrosis, tissue edema, and an increase of IgG4 in the blood. This disease was singled out as an independent nosological unit only in 2001. The incidence is less than 1 in 100,000 people per year. Almost any organ can be affected in IgG4-related disease. The association of Riedel's thyroiditis with IgG4 was established in 2010. Riedel's thyroiditis is an extremely rare inflammatory disease of the thyroid gland, which diagnosis is complicated by an atypical course and the absence of characteristic symptoms. Less than 300 clinical cases of the disease have been described in the world, only two from them were in children. This article presents a clinical case of a 6-year-old boy with Riedel's thyroiditis.

## АКТУАЛЬНОСТЬ

Тиреоидит Риделя (ТР) — это редкое воспалительное заболевание щитовидной железы (ЩЖ), проявляющееся ее постепенным увеличением и замещением соединительной тканью, что сопровождается компрессией близлежащих органов [1].

Впервые ТР описан Бернхардом Риделем в 1896 г. [2]. ТР проявляется прогрессирующим фиброзом, что приводит к разрушению фолликулярных клеток и полному замещению ткани щитовидной железы, вызывая ее уплотнение. Соединительная ткань может распространяться за пределы капсулы ЩЖ, прорастая в окружающие структуры, в том числе сосуды, паращитовидные железы, трахею, пищевод. Клинически для заболевания характерны: болевой синдром, «каменистая» плотность железы, одышка, дисфагия, охриплость голоса, гипотиреоз, гипопаратиреоз [3]. Однако на ранних стадиях заболевания клинические проявления могут отсутствовать [1][3].

Этиология ТР до конца не ясна, но преобладание IgG4-позитивных плазматических клеток в гистологических образцах ЩЖ при ТР позволило включить данное заболевание в структуру IgG4-связанных заболеваний [4].

IgG4-связанное заболевание было впервые описано в 2001 г. у 20 пациентов со склерозирующим панкреатитом, у которых наблюдался повышенный уровень IgG4 в сыворотке крови [5]. Впоследствии ряд заболеваний, имеющих сходные клинические и лабораторные особенности, включая болезнь Микулича, аутоиммунный панкреатит, холецистит, склерозирующий холангит, гипофизит, тиреоидит Риделя, опухоль Кюттнера, интерстициальный пневмонит, интерстициальный нефрит, ретроперитонеальный фиброз и аортит, были объединены в одно понятие — «IgG4-связанные заболевания» [6–10].

Впервые тиреоидит Риделя был отнесен к IgG4-связанным заболеваниям Dahlgren M. и соавт. в 2010 г., когда у 3 пациентов в послеоперационном материале щитовидной железы с помощью иммуногистохимического исследования были обнаружены IgG4-позитивные плазматические клетки [7].

В отечественной литературе первая публикация, посвященная лечению IgG4-связанных заболеваний, датирована 2016 г. и принадлежит Соколу Е.В. и соавт. [11]. В 2017 г. Юкина М.Ю. и соавт. опубликовали литературный обзор, посвященный аутоиммунной эндокринной патологии, связанной с IgG4, а именно заболеваниям гипофиза, поджелудочной железы и щитовидной железы [12]. В 2020 г. Румянцевым П.О. и соавт. опубликован литературный обзор, включивший в себя вопросы этиологии, классификации, иммунопатогенеза, клинической картины, диагностики и лечения IgG4-связанных заболеваний [13].

IgG4-связанное заболевание может поражать практически любой орган, поэтому симптомы зависят от области поражения, а тяжесть заболевания может варьировать. У 60–90% пациентов наблюдается поражение двух и более органов [14]. Заболевание может манифестировать одновременно в нескольких органах или постепенно, вовлекая один орган за другим, что требует тщательного наблюдения за пациентом [13].

У детей преимущественно поражается один орган, чаще других встречается орбитопатия, реже заболевание является системным. Описаны случаи аутоиммунного панкреатита 1 типа, сиалоаденита, заболевания легких, лимфаденопатии, склерозирующего холангита, тиреоидита Риделя, дакриоаденита, мезентерита, гипертрофического пахименингита [15].

Преимущественно клинические проявления IgG4-связанного заболевания носят подострый характер из-за прогрессирующего увеличения органов, и в ряде случаев заболевание выявляется случайно во время диагностических исследований. В 40% случаев у пациентов с IgG4-связанным заболеванием имеется отягощенный аллергологический анамнез (бронхиальная астма, аллергический ринит, атопический дерматит) [13][14].

Патогенез IgG4-связанных заболеваний до конца не известен, специфические аутоантитела не выявлены. К настоящему моменту с уверенностью можно утверждать, что IgG4 в патогенезе IgG4-связанного заболевания не является «центральным игроком» и сам по себе не участвует в тканевом воспалении. Однако IgG4 может активировать цитотоксические Т-лимфоциты, которые представляют основу тканевого воспалительного инфильтрата при IgG4-связанном заболевании. В фазу воспаления происходит клональная экспансия патогенных В-клеток и цитотоксических Т-клеток, которые накапливаются в тканях и секретируют профибротические молекулы (цитокины, хемокины), одновременно привлекая другие клетки (моноциты, миофибробласты, дендритные клетки, макрофаги) в очаг воспаления и способствуют развитию фиброза. В дальнейшем лимфоцитарные инфильтраты замещаются соединительной тканью, что в конечном итоге приводит к нарушению функции пораженного органа [16][17].

В 2011 г. группа японских исследователей из разных областей медицины во главе с Umehara предложила критерии диагностики IgG4-связанных заболеваний [18]. После этого в мире было зарегистрировано множество случаев IgG4-связанных заболеваний, диагностированных по данным критериям. Вместе с тем в клинической практике возник ряд трудностей, включая проведение биопсии органов и оценку уровня сывороточного IgG4 [18].

Через 9 лет, в 2020 г., учитывая многолетний клинический опыт, Umehara и соавт. предложили обновленные критерии комплексной диагностики [19].

Критерии диагностики IgG4-связанных заболеваний делятся на 3 группы [19].

При наличии всех трех критериев диагноз «IgG4-связанное заболевание» сомнений не вызывает, при положительных 1 и 3 критериях диагноз вероятен, при положительных 1 и 2 критериях — диагноз возможен. У пациентов с вероятным или возможным диагнозом и наличием органоспецифических симптомов заболевания диагноз «IgG4-связанное заболевание» считается установленным [19].

Повышенный уровень IgG4 в сыворотке крови выше нормативных значений наблюдается у 70–80% пациентов. Однако данный критерий неспецифичен: повышенный уровень IgG4 может наблюдаться у 5% людей без IgG4-связанного заболевания, а также у 10% пациентов со злокачественными новообразованиями поджелудочной железы, инфекционными и воспалительными заболеваниями. Вовлечение нескольких органов связано с более высокой концентрацией IgG4 в крови. Нормальные уровни IgG4 не исключают наличие IgG4-связанного заболевания. При IgG4-связанном заболевании титры сывороточных ANA (антиядерных антител) положительны в 50% случаев, и повышенный ревматоидный фактор выявляется у 20% пациентов [14].

## КЛИНИЧЕСКИЙ СЛУЧАЙ

Мальчик 6 лет впервые поступил в ГНЦ РФ ФГБУ «НМИЦ эндокринологии» в сентябре 2022 г. с жалобами на узловое образование в щитовидной железе (ЩЖ), выявленное случайно при обследовании по поводу рецидивирующей ангины. По данным УЗ-исследования ЩЖ, проведенном по месту жительства, в верхней половине левой доли выявлено образование с гипоэхогенными включениями и микрокальцинатами.

При поступлении у пациента не было жалоб на боль в области шеи, пальпаторно узловое образование не определялось.

По данным УЗ-исследования ЩЖ, в верхней трети левой доли визуализировалось образование размерами 1,6х1,0х0,9 см с нечеткими контурами, смешанной структуры, преимущественно пониженной эхогенности с умеренным кровотоком по периферии и гиперэхогенными включениями от 0,1 до 0,5 см, EU-TIRADS 5 (рис. 1), объем ЩЖ составлял 3,4 см³. По ходу сосудистых пучков определялись множественные лимфатические узлы диаметром до 0,6 см без видимых изменений.

**Figure:**
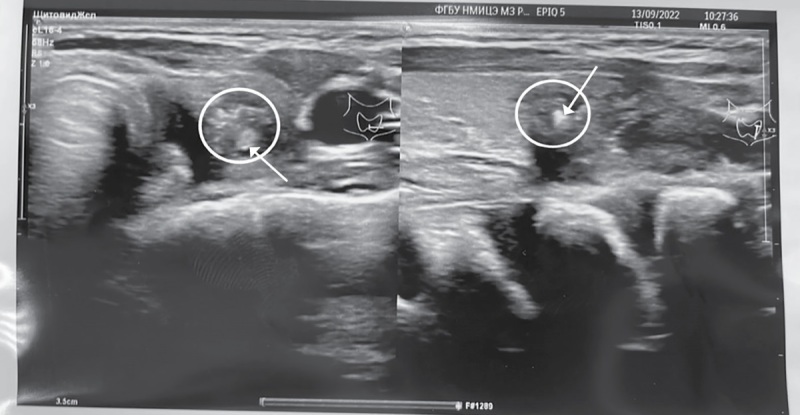
Рисунок 1. Узел в левой доле щитовидной железы (обведен) с гиперэхогенными включениями от 0,1 до 0,5 см (указаны стрелками). Figure 1. Nodule in the left lobe of the thyroid gland (circled) with hyperechoic inclusions from 0.1 to 0.5 cm (indicated by arrows).

В гормональном профиле отмечался эутиреоз: ТТГ — 2,37 мМЕ/л (0,64–5,76), свТ4 — 14,73 пмоль/л (11,2–18,6), свТ3 — 5,6 пмоль/л (2,5–9,2). Уровень кальцитонина — 3,29 пг/мл (0–11,6), титр АТ ТПО — 0,33 МЕ/мл (0–5,6). В общем анализе крови отсутствовали признаки воспаления: лейкоциты — 7,14х⁹ кл/л (5–12х⁹), СОЭ — 10 мм/час (2–15).

По данным тонкоигольной аспирационной биопсии узла, обнаружено большое количество гигантских многоядерных клеток и укрупненных клеток фолликулярного эпителия щитовидной железы с признаками атипии, с напластованиями ядер, лежащих преимущественно разрозненно, что свидетельствовало о фолликулярном образовании, подозрительном в отношении злокачественного (Bethesda-V).

Учитывая полученные результаты исследований, мальчик был переведен в хирургическое отделение для проведения оперативного лечения.

При интраоперационной ревизии левая доля ЩЖ была увеличена, плотная, хрящевидной консистенции, белесоватого цвета, бугристая, в ее составе было узловое образование 1,5 см (рис. 2А), с прорастанием в окружающие претрахеальные мышцы и гортань. Изменения в левой доле были расценены как инвазивный рак щитовидной железы с прорастанием в окружающие мягкие ткани шеи. Операция проходила с техническими сложностями ввиду сращения левой доли ЩЖ с пищеводом. Правая доля ЩЖ была эластической консистенции, обычного цвета (рис. 2Б). Паратрахеальные лимфоузлы центральной зоны были увеличены, овоидной формы, макроскопически не изменены. Выполнена тиреоидэктомия с вовлеченными в процесс окружающими мягкими тканями (рис. 3), центральная лимфаденэктомия.

**Figure:**
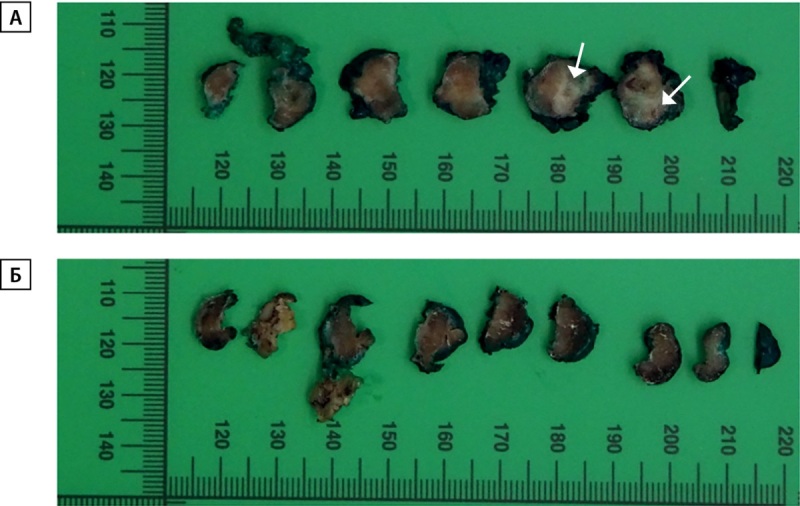
Рисунок 2. А. Макропрепарат левой доли щитовидной железы. Стрелками указан очаг фиброзных изменений.Б. Макропрепарат правой доли щитовидной железы. Структура не изменена. Figure 2. A. Macropreparation of the left lobe of the thyroid gland. Arrows indicate the focus of fibrotic changes. Б. Macropreparation of the right lobe of the thyroid gland. The structure has not been changed.

**Figure:**
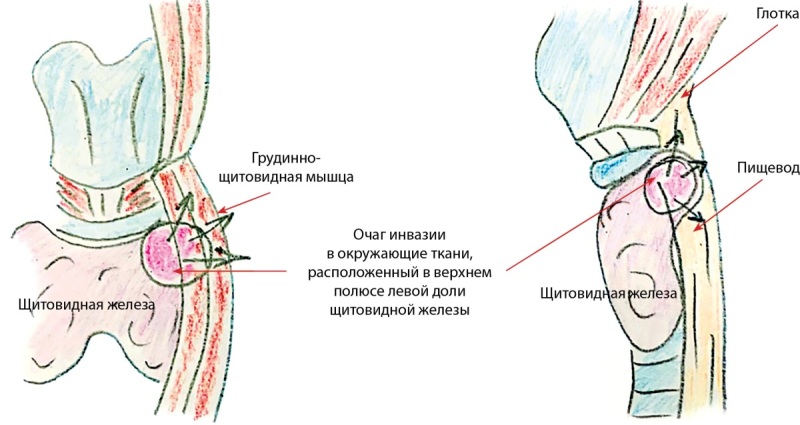
Рисунок 3. Схематичное изображение прорастания фиброзной ткани в окружающие структуры шеи. Figure 3. Schematic representation of the ingrowth of fibrous tissue into the surrounding structures of the neck.

При морфологическом исследовании в левой доле щитовидной железы данных за опухолевый рост не получено, обнаружен обширный участок фиброза с наличием гранулематозного воспаления с гигантскими многоядерными клетками, лимфоцитами с примесью нейтрофилов, а также элементы участка протока с щелевидным просветом, выстланного многорядным эпителием среди лейкоцитарного инфильтрата (рис. 4).

**Figure:**
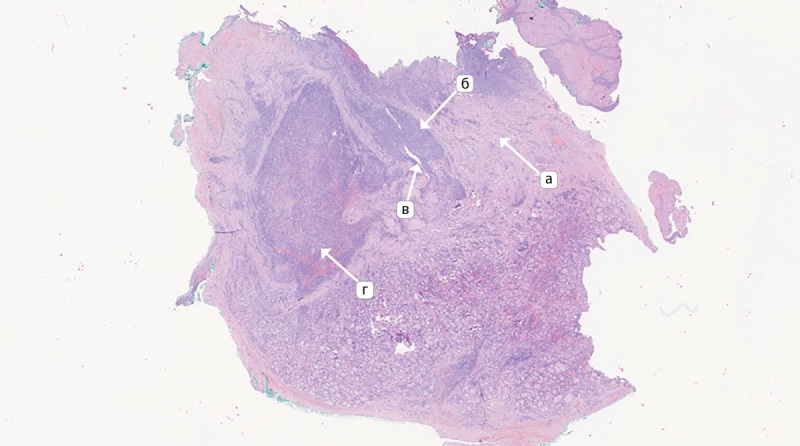
Рисунок 4. Микроскопическое исследование операционного материала левой доли щитовидной железы: а — фиброзная ткань; б — участок гранулематозного воспаления; в — незаращенный щито-язычный проток; г — инфильтрация гигантскими многоядерными клетками, лимфоцитами с примесью нейтрофилов. Окраска гематоксилином и эозином х50. Figure 4. Microscopic examination of the surgical material of the left lobe of the thyroid gland: а — fibrous tissue; б — area of granulomatous inflammation; в — unfused thyroglossal duct; г — infiltration of giant multinucleated cells, lymphocytes with an admixture of neutrophils. Staining with hematoxylin and eosin x50.

Фиброзная ткань распространялась на прилежащие скелетные мышцы (рис. 5).

**Figure:**
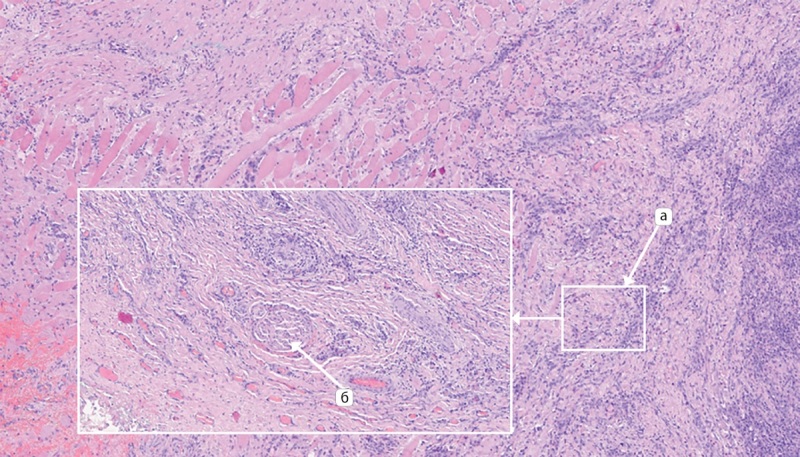
Рисунок 5. Микроскопическое исследование операционного материала, пораженный участок скелетной мышцы: а — фиброзная ткань; б — облитерирующий флебит. Окраска гематоксилином и эозином х100. Figure 5. Microscopic examination of the surgical material, the affected area of skeletal muscle: а — fibrous tissue; б — obliterating phlebitis.Hematoxylin and eosin staining x100

Правая доля щитовидной железы состояла из разнокалиберных фолликулов, выстланных обычным фолликулярным эпителием.

В связи с наличием обширного фиброза с гранулематозным воспалением по результатам послеоперационного морфологического исследования ткани левой доли ЩЖ и отсутствием клеточной атипии, диагностированной при тонкоигольной аспирационной биопсии, было проведено иммуногистохимическое исследование с антителами к макрофагам (CD68), плазматическим клеткам (CD138), IgG, IgG4.

В области фиброзно-воспалительных изменений левой доли ЩЖ было обнаружено обилие плазматических клеток (CD138 позитивных), экспрессирующих IgG4. Отмечалось преобладание IgG4-позитивных клеток в инфильтрате, соотношение IgG4-позитивных плазматических клеток к IgG-позитивным плазматическим клеткам более 50% (рис. 6).

**Figure:**
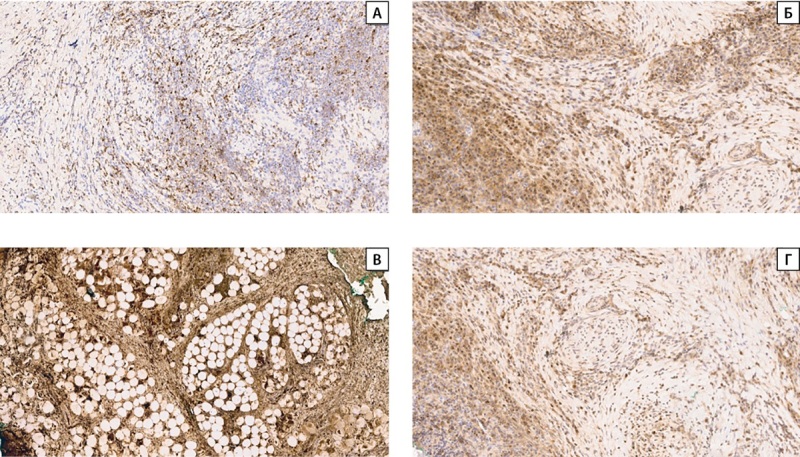
Рисунок 6. Иммуногистохимическое исследование операционного материала с антителами CD68, CD138, IgG, IgG4, выявлена позитивная реакция: А — CD68 (макрофаги). Увеличение х50; Б — CD138 (плазматические клетки). Увеличение х50; В — IgG-позитивные плазматические клетки. Увеличение х50; Г — IgG4-позитивные плазматические клетки. Увеличение х50. Figure 6. Immunohistochemical study of surgical material with antibodies CD68, CD138, IgG, IgG4, a positive reaction was revealed: А — CD68 (macrophages). Magnification x50; Б — CD138 (plasma cells). Magnification x50; В — IgG-positive plasma cells. Magnification x50; Г — IgG4-positive plasma cells. Magnification x50.

В послеоперационном периоде у ребенка был исследован уровень IgG4 крови, который составил 5 мг/дл (10–135).

Таким образом, на основании фиброзно-воспалительных изменений в ЩЖ с распространением на скелетные мышцы, наличия облитерирующего флебита, а также иммуногистохимически подтвержденного преобладания IgG4-позитивных клеток в инфильтрате был поставлен диагноз «IgG4-связанное заболевание — тиреоидит Риделя».

В послеоперационном периоде, на вторые сутки, когда ребенок начал самостоятельно есть, была обнаружена перфорация пищевода, что потребовало наложения гастростомы, которая была удалена на 11-е сутки.

В послеоперационном периоде развился гипопаратиреоз и гипотиреоз, в связи с чем пациенту была назначена терапия препаратами кальция, активной формы витамина D и заместительная терапия левотироксином натрия с положительным эффектом.

## ОБСУЖДЕНИЕ

Представленный клинический случай является первым описанием ТР у ребенка в отечественной литературе.

Первое описание серии клинических случаев ТР было представлено Mitra M. Fatourechi и соавт. в 2010 г. В период с 1976 по 2008 гг. 21 пациент с ТР проходил лечение в клинике Мейо, из них — 17 женщин [20]. Средний возраст на момент постановки диагноза составил 42 года (диапазон от 23 до 65 лет), средний срок наблюдения — 9,5 года (медиана — 4,5 года). Диагноз был поставлен в среднем через 10,2 месяца после появления симптомов и через 23,4 месяца после появления видимых признаков, включая зоб. Основными симптомами заболевания были: ощущение боли у 5 пациентов, симптомы сдавления — у 5, дисфагия — у 7, одышка и нарушение проходимости дыхательных путей — у 9. У 3 пациентов отмечалась лихорадка, а у 6 — паралич голосовых связок. У 10 пациентов, по данным визуализирующих методов исследования, наблюдалось сужение трахеи. При первоначальном осмотре в клинике Мейо у всех пациентов ЩЖ была пальпаторно увеличена, твердая и сращена с окружающими тканями. У 12 из 15 человек титр антител к ТПО был повышен. У 19 пациентов на момент обращения были известны уровни гормонов щитовидной железы: ни у одного пациента не было гипертиреоза, гипотиреоз отмечался у 14 пациентов, эутиреоз был у 5 человек. Восемь пациентов имели экстратиреоидные локализации IgG4-связанных заболеваний: у 4 пациентов был фиброзирующий медиастинит; у 3 был забрюшинный фиброз; у 1 пациента было сочетание фиброза орбиты, фиброза поджелудочной железы и фиброза эпидурального пространства; у 1 пациента имелось подкожное фиброзное образование в грудной клетке в области вентроперитонеального шунта, установленного по поводу гидроцефалии [20].

В самом большом систематическом обзоре A. Zala и соавт. 2020 г. [21] проанализировано 212 клинических случаев ТР за период с 1925 по 2019 гг. Средний возраст пациентов на момент постановки диагноза составил 47 лет, среди которых преобладали женщины (81%). Наиболее распространенными этническими группами были европеоиды (56%) и азиаты (33%). Данные о функции ЩЖ были известны у 150 пациентов: у 46% отмечался гипотиреоз, эутиреоз был у 44%, гипертиреоз был выявлен в 7% случаев, субклинический гипотиреоз был у 2%, субклинический гипертиреоз — у 1% пациентов. Среди частых клинических симптомов отмечались отек шеи (у 162 из 182 пациентов), одышка (у 83 из 167), боль в шее (у 64 из 155), хриплый голос (у 66 из 169), также часто встречались симптомы обструкции верхних дыхательных путей в виде стридора (у 44 из 144 пациентов) и дисфагии (у 97 из 153). Уровень антител к ЩЖ был исследован у 92 человек: антитела к ТПО были повышены у 43% пациентов, антитела к ТГ были положительны у 27%, антитела к рецептору ТТГ были повышены у 2 человек. Среди пациентов, у которых был исследован С-реактивный белок, его повышение отмечалось в 72% случаев, СОЭ была повышена в 97% случаев [21].

В случаях, когда был клинически заподозрен ТР, подтверждение осуществлялось посредством открытой биопсии, тотальной тиреоидэктомии или гемитиреоидэктомии. Подробное гистологическое описание послеоперационного материала было доступно в 179 случаях, при этом фиброзная ткань присутствовала во всех образцах. Другими общими признаками были лимфоцитарная инфильтрация (98%) и облитерирующий флебит (43%) [21].

Лечение проводилось хирургически и медикаментозно с помощью иммунодепрессантов. Хирургическое вмешательство потребовалось 161 пациенту. Наиболее частым методом была тотальная тиреоидэктомия (34%), гемитиреоидэктомия проведена в 12% случаев, трахеостомия — в 10%. Глюкокортикоиды применялись у 121 пациента, продолжительность терапии составила от 3 дней до 60 месяцев. Тамоксифен применялся в 28 случаях, азатиоприн — в 4 случаях, ритуксимаб — в 3 случаях, также был один случай применения микофенолата мофетила [21].

Медиана периода наблюдения составила 12 месяцев (0,1–566 месяцев), 10 пациентов умерли, 2 смерти были связаны с прогрессированием ТР и системным фиброзом [21].

В описательном обзоре Carsote M. и соавт., опубликованном в июне 2023 г., проанализировано 66 клинических случаев ТР с 2019 по 2023 гг. [22]. Было отмечено, что в большинстве клинических случаев наличие ТР предполагалось на этапе постановки диагноза, что свидетельствует о возросшей осведомленности врачей о данном заболевании [22].

Zakeri H. и соавт. в 2011 г. описали клинический случай ТР у 17-летнего подростка, обратившегося с жалобами на объемное образование и легкую боль в области шеи. УЗИ шеи выявило неоднородное твердое образование в левой доле ЩЖ со смещением сосудов шеи и грудино-ключично-сосцевидной мышцы, множественную лимфаденопатию в переднем треугольнике шеи слева. В гормональном профиле был выявлен тиреотоксикоз (Т4 — 18,3 пмоль/л, Т3 — 265 пмоль/л, ТТГ — менее 0,1 мМЕ/л). При тонкоигольной биопсии выявлена инфильтрация мононуклеарными клетками и фиброз. В ходе открытой биопсии обнаружено образование бело-серого цвета с вовлечением окружающих тканей и мышц. При гистологическом исследовании описана инфильтрация паренхимы ЩЖ мононуклеарными клетками, фиброз, деструкция с вовлечением грудино-ключично-сосцевидной мышцы. Ткань ЩЖ была полностью замещена соединительной тканью (тироциты не определялись). Пациент получал пропранолол и преднизолон в течение трех месяцев. После месяца лечения состояние его улучшилось, размер образования уменьшился, структура грудино-ключично-сосцевидной мышцы, по данным УЗИ, нормализовалась, сохранялась неоднородность и гипоплазия левой доли [23].

В 2020 г. Dhalapathy Sadacharan и соавт. опубликовали серию из 6 клинических случаев пациентов с ТР, диагностированного в период с 2011 по 2019 гг. на юге Индии [24]. Один из них был у 5-летнего мальчика, у которого на момент постановки диагноза отмечались болезненный отек на передней поверхности шеи и одышка [24]. Результат тонкоигольной аспирационной биопсии выявил наличие воспалительных мононуклеарных клеток и миофибробластов. Мальчику было проведено оперативное лечение (гемитиреоидэктомия слева) и удаление фиброзного образования, прорастающего в мышцы. Вместе с левой долей щитовидной железы были удалены верхняя и нижняя паращитовидные железы слева, гипокальциемия была купирована через 3 месяца после операции. Период наблюдения составил 1 год, прогрессии заболевания не отмечалось, других локализаций IgG4-связанных заболеваний не выявлено [24].

Для диагностики ТР могут быть полезны компьютерная томография, на изображении которой определяется гиподенсивная ткань, не накапливающая йодсодержащий контраст, или магнитно-резонансная томография, на которой следует ожидать получение гипоинтенсивного изображения как на взвешенных изображениях T1, так и на T2 [4].

В большинстве описанных случаев ТР у пациентов не отмечалось повышение уровня IgG4 в сыворотке крови [4]. В одном из исследований предложен органоспецифический критерий для диагностики ТР — соотношение IgG4-позитивных плазматических клеток к IgG-позитивным плазматическим клеткам более 20% [25].

Из-за обширного фиброза тонкоигольная аспирационная биопсия чаще всего не информативна, так как в биоптате отсутствуют фолликулярные клетки, но могут быть выявлены признаки воспалительного процесса, фиброзная ткань, миофибробласты или признаки, соответствующие фолликулярному новообразованию [4], и для точной постановки диагноза ТР и дифференциальной диагностики со злокачественным новообразованием необходима хирургическая открытая биопсия [21].

Дифференциальная диагностика ТР является сложной задачей. Ее следует проводить с тиреоидитом Хашимото и папиллярным раком щитовидной железы, при этом выраженность фиброза при них меньше, чем при ТР, и окружающие ткани не вовлекаются в процесс. При анапластическом раке щитовидной железы, помимо фиброза и некроза, обнаруживаются атипичные веретенообразные клетки [4].

В настоящее время не существует стандартов лечения ТР и рандомизированных контролируемых исследований, доказывающих эффективность какой-либо определенной схемы лечения. Методом выбора является лечение препаратами глюкокортикостероидов. При их неэффективности рекомендовано применение тамоксифена, микофенолата мофетила или ритуксимаба [1]. Хирургическое вмешательство при ТР оправдано в случаях компрессии дыхательных путей и необходимости забора биоматериала для гистологического исследования [3].

## ЗАКЛЮЧЕНИЕ

Представленный нами случай тиреодита Риделя является уникальным в силу раннего возраста ребенка, отсутствия каких-либо характерных клинических признаков, позволяющих диагностировать заболевание на дооперационном этапе.

Тиреоидит Риделя является чрезвычайно редким заболеванием, диагностика которого представляет трудную клиническую задачу для врачей, в связи с тем, что имеет неспецифическую клиническую картину, ультразвуковая диагностика и тонкоигольная аспирационная биопсия не имеют диагностической значимости, исследование уровня IgG4 в крови не показательно при ТР, а открытая биопсия не входит в рутинную практику исследования заболеваний ЩЖ.

## ДОПОЛНИТЕЛЬНАЯ ИНФОРМАЦИЯ

Источники финансирования. Исследование было проведено в рамках государственного задания «Молекулярно-генетические маркеры стратификации риска прогрессирования/рецидива рака щитовидной железы» №123021000039-3.

Конфликт интересов. Авторы декларируют отсутствие явных и потенциальных конфликтов интересов, связанных с содержанием настоящей публикации.

Согласие пациента. Авторы настоящей статьи получили письменное разрешение от упоминаемых в статье пациентов на публикацию их медицинских данных в журнале «Проблемы эндокринологии».

Участие авторов. Зубкова Н.А., Бондаренко С.П. — анализ данных, интерпретация результатов, подготовка финальной версии статьи; Колодкина А.А., Безлепкина О.Б. — редактирование текста, внесение ценных замечаний; Урусова Л.С., Бровин Д.Н., Аникиев А.В. — анализ данных, интерпретация результатов, написание статьи. Все авторы одобрили финальную версию статьи перед публикацией, выразили согласие нести ответственность за все аспекты работы, подразумевающую надлежащее изучение и решение вопросов, связанных с точностью или добросовестностью любой части работы.
